# Association of CTLA-4 Gene Variants with Response to Therapy and Long-term Survival in Metastatic Melanoma Patients Treated with Ipilimumab: An Italian Melanoma Intergroup Study

**DOI:** 10.3389/fimmu.2017.00386

**Published:** 2017-04-12

**Authors:** Paola Queirolo, Beatrice Dozin, Anna Morabito, Barbara Banelli, Patrizia Piccioli, Cristiana Fava, Claudio Leo, Roberta Carosio, Stefania Laurent, Vincenzo Fontana, Pier Francesco Ferrucci, Chiara Martinoli, Emilia Cocorocchio, Angelo Battaglia, Paolo A. Ascierto, Mariaelena Capone, Ester Simeone, Federica De Galitiis, Elena Pagani, Gian Carlo Antonini Cappellini, Paolo Marchetti, Michele Guida, Stefania Tommasi, Mario Mandalà, Barbara Merelli, Pietro Quaglino, Paolo Fava, Massimo Guidoboni, Massimo Romani, Francesco Spagnolo, Maria Pia Pistillo

**Affiliations:** ^1^Department of Medical Oncology, IRCCS AOU San Martino-IST-Istituto Nazionale per la Ricerca sul Cancro, Genova, Italy; ^2^Unit of Clinical Epidemiology, IRCCS AOU San Martino-IST-Istituto Nazionale per la Ricerca sul Cancro, Genova, Italy; ^3^Unit of Tumor Epigenetics, IRCCS AOU San Martino-IST-Istituto Nazionale per la Ricerca sul Cancro, Genova, Italy; ^4^Department of Health Sciences, University of Genova, Genova, Italy; ^5^Unit of Cellular Biology, IRCCS AOU San Martino-IST-Istituto Nazionale per la Ricerca sul Cancro, Genova, Italy; ^6^Intergruppo Melanoma Italiano (IMI) and Department of Internal Medicine, University of Genova, Genova, Italy; ^7^Oncology of Melanoma Unit, European Institute of Oncology, Milan, Italy; ^8^Melanoma, Cancer Immunotherapy and Innovative Therapy Unit, Istituto Nazionale Tumori Fondazione ‘G. Pascale’, Naples, Italy; ^9^Istituto Dermopatico dell’Immacolata IDI-IRCCS, Rome, Italy; ^10^Medical Oncology, Sant’Andrea Hospital, Sapienza University of Rome, Rome, Italy; ^11^Department of Medical Oncology and Molecular Genetics Laboratory, IRCCS Istituto Tumori “Giovanni Paolo II”, Bari, Italy; ^12^Unit of Medical Oncology, Department of Oncology and Hematology, Papa Giovanni XXIII Hospital, Bergamo, Italy; ^13^Dermatologic Clinic, Department of Medical Sciences, University of Turin, Turin, Italy; ^14^Immunotherapy and Cell Therapy, IRCCS-IRST, Meldola, Italy

**Keywords:** CTLA-4 variants, melanoma, ipilimumab, best overall response, overall survival, predictive/prognostic factor

## Abstract

Ipilimumab (IPI) blocks CTLA-4 immune checkpoint resulting in T cell activation and enhanced antitumor immunity. IPI improves overall survival (OS) in 22% of patients with metastatic melanoma (MM). We investigated the association of CTLA-4 single nucleotide variants (SNVs) with best overall response (BOR) to IPI and OS in a cohort of 173 MM patients. Patients were genotyped for six CTLA-4 SNVs (−1661A>G, −1577G>A, −658C>T, −319C>T, +49A>G, and CT60G>A). We assessed the association between SNVs and BOR through multinomial logistic regression (MLR) and the prognostic effect of SNVs on OS through Kaplan–Meier method. Both −1577G>A and CT60G>A SNVs were found significantly associated with BOR. In particular, the proportion of responders was higher in G/G genotype while that of stable patients was higher in A/A genotype. The frequency of patients experiencing progression was similar in all genotypes. MLR evidenced a strong downward trend in the probability of responsiveness/progression, in comparison to disease stability, as a function of the allele A “dose” (0, 1, or 2) in both SNVs with reductions of about 70% (G/A vs G/G) and about 95% (A/A vs G/G). Moreover, −1577G/G and CT60G/G genotypes were associated with long-term OS, the surviving patients being at 3 years 29.8 and 30.8%, respectively, as compared to 12.9 and 14.4% of surviving patients carrying −1577G/A and CT60G/A, respectively. MM patients carrying −1577G/G or CT60G/G genotypes may benefit from IPI treatment in terms of BOR and long-term OS. These CTLA-4 SNVs may serve as potential biomarkers predictive of favorable outcome in this subset of patients.

## Introduction

Ipilimumab (IPI) is a human monoclonal antibody targeting the immune-checkpoint molecule CTLA-4 (cytotoxic T lymphocyte antigen-4), which is expressed on activated effector T cells (Teff cells) and regulatory T cells (Tregs). CTLA-4 negatively regulates Teff cell activation through inhibition of cell proliferation, IL2 production, and cell-cycle progression (1, 2) upon binding to B7 ligands (CD80/CD86) expressed by the antigen-presenting cells. IPI inhibits the CTLA-4/B7 interaction, thus promoting costimulation and proliferation of Teff cells as well as their infiltration into the tumor (3, 4).

Ipilimumab was approved for the treatment of metastatic melanoma (MM) following the demonstration of a statistically significant improvement in overall survival (OS) in two randomized phase III trials in pre-treated (5) and treatment-naïve (6) MM patients. IPI may achieve clinical benefit in terms of long-lasting disease control and long-term survival in ≈20% of patients (5, 7).

The increasing number of treatment options available (including targeted therapies and other immune-checkpoint inhibitors) and the evidence that IPI may achieve a relevant clinical benefit (i.e., durable response to treatment and long-term survival) in a small subset of patients highlight the need to investigate predictive biomarkers that identify this subset of patients.

In this context, several potential biomarkers have been proposed including serum lactate dehydrogenase (LDH) (8), peripheral blood lymphocyte count (8), neutrophil-to-lymphocyte ratio (9), intratumoral immune cell infiltration (10), mutational load (11), and other parameters underlying cancer-immune cell interactions (12). However, despite some correlations with response to IPI, these predictive biomarkers have not yet proved sufficiently robust to be used clinically (13).

Single-nucleotide variants (SNVs) of CTLA-4 gene may represent novel predictive biomarkers in IPI-treated MM patients for their influence on the host immune response through alteration of CTLA-4 expression levels and function in T cells, as shown primarily in autoimmune diseases (14).

In particular, they can affect the transcriptional efficiency of CTLA-4 gene (14–16), CTLA-4 processing and intracellular/surface transport (17), the interaction between CTLA-4 and CD80 ligand (18), and the levels of CTLA-4 soluble isoform (14). Thus, it is reasonable that CTLA-4 gene variants may have a role also in the response to a CTLA-4-based immunotherapy.

Indeed, CTLA-4 SNVs have shown some implications both in clinical response (19) and OS (20) in MM patients treated with anti-CTLA-4 therapy. In a previous pilot study, we were able to identify two out of six specific CTLA-4 SNVs (namely −1577G>A and CT60G>A) as having a potential role in OS and response to IPI (20). This preliminary study, conducted on a limited cohort of patients, was mainly a hypothesis generating investigation that was further assessed in the present multicentre study. We here report on the genotype distribution of six functionally relevant CTLA-4 variants (SNVs −1661A>G, −1577G>A, −658C>T, −319C>T, +49A>G, CT60G>A) in a cohort of 173 MM patients treated with IPI and on their association with response to IPI treatment and OS.

## Materials and Methods

### Patient Features, IPI Treatment, and Response Evaluation

The present multicenter retrospective study included 173 patients, aged ≥16, presenting stage IV melanoma with 0–2 Eastern Cooperative Oncology Group performance status, who failed to respond or were intolerant to at least one prior systemic treatment and no other therapeutic option.

Among them, 122 were enrolled between July 2010 and January 2012 within an expanded access program [EAP; Ref. (21)] aimed at the compassionate use of IPI. Additional 51 patients were enrolled between February 2012 and November 2013 after IPI use in Italy had been approved by the AIFA (Agenzia Italiana del Farmaco, Rome, Italy). Twelve Italian centers, members of the Italian Melanoma Intergroup (IMI) network, participated in patient recruitment. The observation period ended by June 30th, 2015.

Enrolled patients were treated intravenously with IPI (3 mg/kg) every 3 weeks (induction dose) for a maximum of four doses. Patients treated within the EAP were eligible to receive re-treatment with IPI 3 mg/kg every 3 weeks for four doses if they progressed after achieving disease control during induction therapy.

Clinical response was assessed as the best overall response (BOR) and classified by the oncologist according to immune-related response criteria (22) as immune-related complete response (irCR) or partial response (irPR) observed at any time during the study. The first scheduled tumor assessment was at week 12 following IPI initiation. Immune-related stable disease (irSD) was defined as failure to meet criteria for irCR or irPR, together with absence of progressive disease (irPD).

One hundred unrelated healthy Italian subjects (Transfusion Service, IRCCS AOU San Martino-IST, Genova, Italy), matched for patient gender and age at the time of first IPI treatment, were analyzed as controls, upon written informed consent.

### CTLA-4 Genotyping

Genomic DNA was extracted from whole peripheral blood samples as already described (23).

Genotyping of CTLA-4 SNVs −1661A>G (rs4553808), −1577G>A (rs11571316), −658C>T (rs11571317) in the 5′UTR and in the promoter region, and CT60G>A (rs3087243), present in the 3′UTR of the gene, was performed by previously described multiple pyrosequencing (PSQ) methods (23). Briefly, the EpMotion5070 liquid handling station (Eppendorf, Milan, Italy) was utilized to assemble the PSQ-PCR reactions in a final volume of 50 µl containing 200 μmol/LdNTPs, 1× GeneAmp buffer (1.5 mM MgCl_2_), 1.25 U of Immolase Hot Start polymerase (Bioline, Milan, Italy), and 0.3 µM of the PCR primer pairs specific for every SNV. The sequencing reactions were performed with the Pyro Gold reagent kit PSQ 96MA according to the manufacturer using a PSQ96MA instrument (Qiagen, Milan, Italy). The SNV genotyping was carried out analyzing multiple (for −1661 and −658 SNVs) and single PSQ reactions (for −1577 and CT60 SNVs). The sequencing analysis was conducted with the PSQ™ 96MA (version 2.02) software.

Genotyping of −319C>T (rs5742909, in the promoter) and +49A>G (rs231775, in the exon 1) SNVs was performed by previously described Tetra-primer Amplification Refractory Mutation System PCR [T-ARMS-PCR; Ref. (24)] and part of the results were validated with the PSQ assay as previously described (25).

### Statistical Analysis

Comparison of SNV genotype and allele frequencies between patients and healthy subjects was performed using the Pearson’s χ^2^ test or the Fisher’s exact test, as appropriate.

Deviation from the Hardy–Weinberg Equilibrium (HWE) was analyzed with the Pearson’s χ^2^ test by using the de Finetti program (http://ihg.gsf.de/cgi-bin/hw/hwa1.pl). A *P*-value <0.05 indicates a lack of HWE.

Patients and disease characteristics were explored using descriptive statistics and expressed as relative frequencies (percentages) for discrete variables and medians for continuous variables.

The primary endpoint was the relationship between each SNV and BOR. In this context, a multinomial logistic regression (MLR) was applied to the three-level clinical response (BOR: irPR + irCR, irSD, and irPD) in order to estimate the predictive role of SNVs while controlling for some important characteristics (age, gender, visceral metastases, LDH levels, and number of pre-IPI therapies) (26). MLR can be considered as an extension of the more widely used logistic regression modeling for dichotomous outcome (i.e., responders vs non-responders) in that it allows to perform simultaneously two binary comparisons: irPR + irCR vs irSD and irPD vs irSD. Within each binary comparison, odds ratio (OR) point estimate, with 95% confidence interval (95% CI), was computed and considered as a relative index of association between SNVs and the clinical outcome.

The secondary endpoint was OS as estimated from the date of first IPI cycle to the date of last contact or death from any cause. OS was analyzed using the Kaplan–Meier method and differences between groups were assessed by the log-rank test.

All statistical analyses were carried out using the SPSS package (version 20.0 for Windows). Statistical significance was accepted for any two-sided *P*-value < 0.05.

## Results

### Patient Features, Disease Characteristics, and Treatment Regimes

Overall, the present study included 173 MM patients enrolled from July 2010 to November 2013. The observation period ended by June 30th, 2015. The primary melanoma subtype, based on tumor location, was cutaneous (*n* = 130, 75.1%), mucosal (*n* = 17, 9.8%), or ocular (*n* = 14, 8.1%) with a distribution in line with that reported in the EAP study (21). For the remaining 12 (6.9%) patients, the primary tumor site was unknown.

Main patient and disease characteristics are summarized in Table 1 together with treatment regimens. By the time of the first IPI cycle, 35 (20.2%) patients had brain metastases, 68 (39.3%) had liver metastases, and 13 (7.5%) had both. Other 81 (46.8%) patients had metastases at sites different from brain and/or liver. For two (1.2%) patients, the site of metastases was unknown. Overall, more than one-third of all patients (*n* = 60, 34.7%) presented diffused disease with metastases at three or more sites.

**Table 1:** **Patient (*n* = 173) features, disease characteristics, and treatment regimens**.

Characteristic	*n* (%)
Age, years, at first IPI cycle (median, range)	58.9 (26.6–88.2)
Gender	
Male	96 (55.5)
Female	77 (44.5)
Primary melanoma subtype	
Cutaneous	130 (75.1)
Mucosal	17 (9.8)
Ocular	14 (8.1)
Missing	12 (6.9)
ECOG performance status	
0	113 (65.3)
1	51 (29.5)
2	9 (5.2)
Serum LDH	
Normal level	65 (37.6)
≥Upper limit of normal	92 (53.2)
Missing	16 (9.2)
Number of metastasis sites at first IPI cycle	
1	60 (34.7)
2	51 (29.5)
≥3	60 (34.7)
Missing	2 (1.2)
Brain metastases	
Present	35 (20.2)
Absent	136 (78.6)
Missing	2 (1.2)
Liver metastases	
Present	68 (39.3)
Absent	103 (59.5)
Missing	2 (1.1)
Other metastases	
Present	81 (46.8)
Absent	90 (52.0)
Missing	2 (1.2)
Number of IPI cycles administered	
4	130 (75.1)
3	8 (4.6)
2	16 (9.2)
1	19 (11.0)
Number of lines of therapies prior to IPI	
1	114 (65.9)
2	45 (26.0)
≥3	14 (8.1)
Chemotherapy^a^ prior to IPI	
Yes	144 (83.2)
No	29 (16.8)
Immunotherapy^b^ prior to IPI	
Yes	34 (19.7)
No	139 (80.3)
Targeted therapy^c^ prior to IPI	
Yes	23 (13.3)
No	150 (86.7)
Therapy post-IPI^d^	
Yes	69 (39.9)
No	102 (59.0)
Missing	2 (1.1)

*IPI, ipilimumab; ECOG, Eastern Cooperative Oncology Group; LDH, lactate dehydrogenase*.

*^a^Including mainly dacarbazine, temozolomide, and fotemustine*.

*^b^Including mainly interferon alpha and interleukin-2*.

*^c^BRAF/MEK inhibitors including vemurafenib, pimasertib, and dabrafenib*.

*^d^Including IPI, chemotherapy, BRAF/MEK inhibitors, and anti PD-1*.

One hundred and thirty (75.1%) patients received the scheduled four doses of IPI; the remaining 43 (24.9%) patients received three or less doses.

Most patients (*n* = 114, 65.9%) received 1 line of systemic therapy prior to treatment with IPI, while 45 (26.0%) and 14 (8.1%) received 2 or ≥3 lines, respectively. Chemotherapy was the most frequent regimen used (*n* = 144, 83.2%) followed by immunotherapy (*n* = 34, 19.7%) and targeted therapy (*n* = 23, 13.3%). Among the patients receiving immunotherapy, 22 received interferon alone and 12 received chemotherapy associated with interferon and/or interleukin-2. IPI was never administered.

Following IPI therapy, 69 (39.9%) patients among those who experienced disease progression received further systemic treatment, including chemotherapy, BRAF/MEK inhibitors, anti-PD-1, or IPI re-treatment.

### Genotyping and Frequencies of CTLA-4 Variants in MM Patients and Control Subjects

Six CTLA-4 SNVs, −1661A>G, −1577G>A, −658C>T, −319C>T, +49A>G, and CT60G>A, were analyzed in 173 MM patients and 100 healthy control subjects adequately matched for gender and age.

Genotype and allele frequencies are reported in Table 2. As shown, no deviation from the HWE was observed for any SNV and similar frequencies of genotypes and alleles were found in both patient and control groups for all the six CTLA-4 SNVs analyzed.

**Table 2:** **Frequencies of six CTLA-4 gene variants in 173 metastatic melanoma patients and in healthy control subjects**.

	*n* (%)				*n* (frequency)		
Genotypes	Melanoma patients (*n* = 173)	Controls (*n* = 100)	*P*^¶^	Alleles	Melanoma patients (2*n* = 346)	Controls (2*n* = 200)	P*
**−1661A>G**				**−1661A>G**			
A/A	102 (59.0)	58 (58.0)	*0.868*	A	266 (0.77)	154 (0.77)	*1.000*
A/G	62 (35.8)	38 (38.0)		G	80 (0.23)	46 (0.23)	
G/G	9 (5.2)	4 (4.0)					
HWE	*P* = 0.915	*P* = 0.466					
**−1577G>A**				**−1577G>A**			
G/G	49 (28.3)	26 (26.0)	*0.807*	G	186 (0.54)	102 (0.51)	*0.535*
G/A	88 (50.9)	50 (50.0)		A	160 (0.46)	98 (0.49)	
A/A	36 (20.8)	24 (24.0)					
HWE	*P* = 0.761	*P* = 0.997					
**−658C>T**				**−658C>T**			
C/C	139 (80.3)	76 (76.0)	*0.669*	C	310 (0.89)	175 (0.87)	*0.482*
C/T	32 (18.5)	23 (23.0)		T	36 (0.11)	25 (0.13)	
T/T	2 (1.2)	1 (1.0)					
HWE	*P* = 0.917	*P* = *0.607*					
**−319C>T**				**−319C>T**			
C/C	139 (80.3)	81 (81.0)	*0.517*	C	311 (0.90)	179 (0.89)	*0.884*
C/T	33 (19.1)	17 (17.0)		T	35 (0.10)	21 (0.11)	
T/T	1 (0.6)	2 (2.0)					
HWE	*P* = 0.519	*P* = 0.339					
**+49A>G**				**+49A>G**			
A/A	96 (55.5)	55 (55.0)	*0.780*	A	255 (0.74)	149 (0.75)	*0.919*
A/G	63 (36.4)	39 (39.0)		G	91 (0.26)	51 (0.25)	
G/G	14 (8.1)	6 (6.0)					
HWE	*P* = 0.425	*P* = 0.791					
**CT60G>A**				**CT60G>A**			
G/G	42 (24.3)	24 (24.0)	*0.877*	G	173 (0.50)	97 (0.48)	*0.790*
G/A	89 (51.4)	49 (49.0)		A	173 (0.50)	103 (0.52)	
A/A	42 (24.3)	27 (27.0)					
HWE	*P* = 0.748	*P* = 0.848					

Genotyping of −1661A>G, −1577A>G, −658C>T, and CT60G>A was performed by the pyrosequencing (PSQ) method and genotyping of −319C>T and +49A>G was performed by T-ARMS PCR and confirmed by PSQ as described in the Section “Materials and Methods”. Comparison of CTLA-4 genotypic and allelic frequencies between melanoma patients and control subjects was estimated using the Pearson’s χ^2^ test (^¶^*P*-value) and the Fisher’s test (*P-value), respectively. Statistical significance: *P* < 0.05. HWE, Hardy–Weinberg equilibrium tested by χ^2^ test (*P* < 0.05 indicates a lack of HWE).

### Association of CTLA-4 Variant Genotype with Response to IPI

Of the 173 patients, 36 (20.8%) showed irPR or irCR, 16 (9.2%) showed irSD, and 121 (69.9%) showed irPD (Table 3).

**Table 3:** **Association of CTLA-4 −1577G>A and CT60G>A SNV genotypes with best overall response to ipilimumab in metastatic melanoma patients estimated through a multinomial logistic regression modeling**.

CTLA-4 SNV	Genotype	Total	irPR + irCR		irSD		irPD		irPR + irCR vs irSD			irPD vs irSD		
			*N*	(%)	*N*	(%)	*N*	(%)	OR	95% CI	*P*-value	OR	95% CI	*P*-value
−1577G>A	G/G	49	14	(28.6)	1	(2.0)	34	(69.4)	1.00	(Ref.)	<0.010	1.00	(Ref.)	<0.010
	G/A	88	17	(19.3)	6	(6.8)	65	(73.9)	0.24	0.02–2.33		0.26	0.03–2.59	
	A/A	36	5	(13.9)	9	(25.0)	22	(61.1)	0.04	0.01–0.44		0.05	0.01–0.49	
CT60G>A	G/G	42	13	(30.9)	1	(2.4)	28	(66.7)	1.00	(Ref.)	<0.010	1.00	(Ref.)	0.010
	G/A	89	18	(20.2)	6	(6.7)	65	(73.1)	0.28	0.03–2.61		0.31	0.03–2.94	
	A/A	42	5	(11.9)	9	(21.4)	28	(66.7)	0.07	0.01–0.61		0.10	0.01–0.90	
Total per SNV		173	36	(20.8)	16	(9.3)	121	(69.9)	–	–	–	–	–	–

*SNV, single nucleotide variant; irPR, immune-related partial response; irCR, immune-related complete response; irSD, immune-related stable disease; irPD: immune-related progressive disease; OR, odds ratio adjusted for age, gender, visceral metastases, lactate dehydrogenase levels, and number of pre-IPI therapies; 95% CI, 95% confidence interval for OR; P-value, likelihood-based χ^2^ test for heterogeneity; Ref., reference category*.

For both −1577G>A and CT60G>A SNVs, patients showed a similar frequency of irPD, regardless the genotypes, whereas G/G carriers presented a higher rate of irPR + irCR (28.6% for −1577G>A and 30.9% for CT60G>A) as opposite to the A/A carriers who presented a higher rate of irSD (25.0% for −1577G>A and 21.4% for CT60G>A). The G/A carriers presented an intermediate frequency of both irPR + irCR (19.3% for −1577G>A and 20.2% for CT60G>A) and irSD (6.8% for −1577G>A and 6.7% for CT60G>A) (Table 3, left side).

Multinomial logistic regression analysis confirmed the results obtained in descriptive analysis. Considering OR as the relative probability of responsiveness/progression vs stability, we found a generalized reduction in the proportion of responder and progressive patients as the “dose” of allele A increases in both SNVs. In particular, using G/G as a reference category, G/A showed a probability of irPr + irCR which was on average 70% lower than the reference (−1577G>A: OR = 0.24, 95% CI = 0.02–2.33; CT60G>A: OR = 0.28, 95% CI = 0.03–2.61), while A/A experienced a reduction of about 95% (−1577G>A: OR = 0.04, 95% CI = 0.01–0.44; CT60G>A: OR = 0.007, 95% CI = 0.01–0.061). Using the same modeling constraints, namely G/A and A/A vs G/G, a very similar tendency in allele A “dose” was also observed for irPD vs irSD comparison (Table 3, right side).

### Association of CTLA-4 Variant Genotype with OS

Median follow-up was 8.1 months (range 0.9–58 months). Overall, 142 (82.1%) deaths were observed. Among the 31 surviving patients, 28 (90.3%) had completed the scheduled four cycles of IPI.

**Figure 1:**
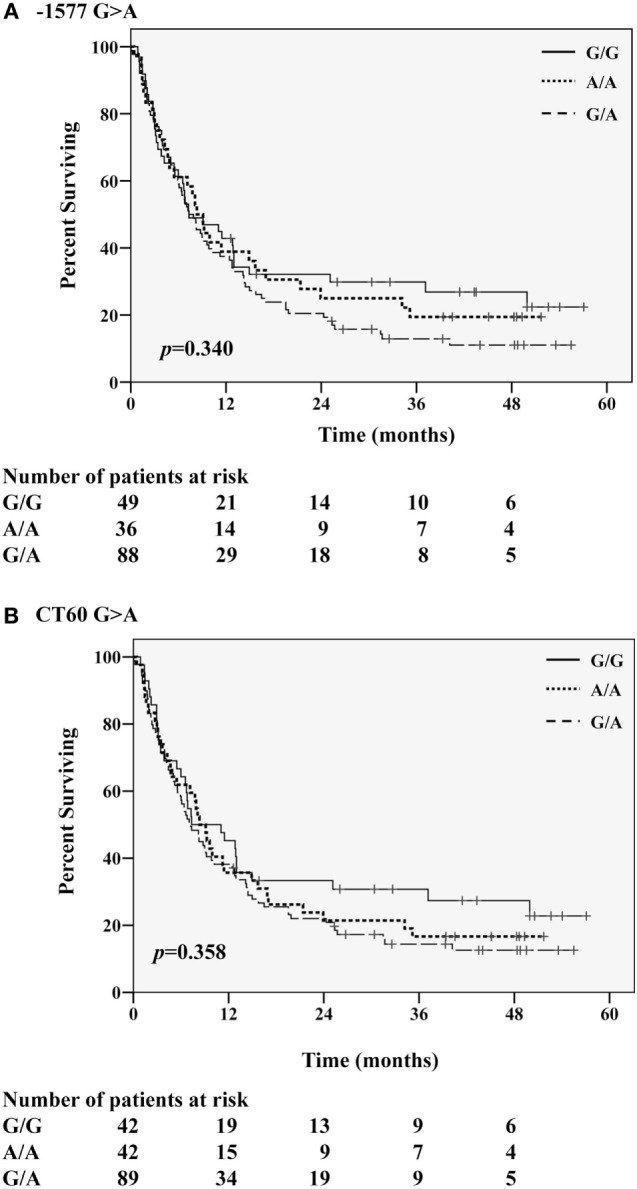
**Kaplan–Meier curves for overall survival according to the homozygous G/G or A/A and the heterozygous G/A genotypes of (A) −1577G>A and (B) CT60G>A CTLA-4 single nucleotide variants**.

Correlation of CTLA-4 variants with OS indicated that two of the six SNVs, namely −1577G>A and CT60G>A, had a noticeable effect on long-term OS. During the first 15 months from the start of IPI treatment, the mortality rate was similar regardless the genotype carried (Figure 1A for −1577G>A and Figure 1B for CT60G>A). Thereafter, not only the survival curves started to stabilize up to more than 4 years but also to separate. Although median OS was similar among all genotypes, a better survival was always observed in patients carrying the G/G genotype: regarding the −1577G>A SNV carriers, their survival rate was remarkably higher as compared to the G/A carriers, being 29.8 vs 12.9%, at 3 years and 26.9 vs 11.1%, at 4 years (Table 4, log rank *p* = 0.172, not shown). The G/G carriers showed a higher survival rate also with respect to the A/A carriers (29.8 vs 19.4%, at 3 years). Similarly, a higher percentage of survivors at 3 years was observed in the CT60G/G carriers as compared to the CT60G/A carriers, being 30.8 vs 14.4% at 3 years (Table 4, log rank *p* = 0.154, not shown). Such difference was maintained at 4 years (27.4 vs 12.6%). The CT60A/A carriers showed a survival rate comparable to that of the CT60G/A carriers at any time point.

**Table 4:** **Median overall survival (OS) and survival rates for metastatic melanoma patients treated with ipilimumab at defined time intervals**.

Genotypes	OS (months)		1 year		2 years		3 years		4 years	
	Median	95% Confidence interval (95% CI)	%	95% CI	%	95% CI	%	95% CI	%	95% CI
**−1577G>A**										
G/G (*n* = 49)	7.3	1.5–13.2	42.9	29.0–56.8	32.1	19.0–45.2	29.8	16.9–42.7	26.9	13.9–39.8
G/A (*n* = 88)	7.3	5.2–9.5	37.5	29.4–45.6	20.5	13.8–27.2	12.9	7.1–18.7	11.1	5.5–16.7
A/A (*n* = 36)	8.3	6.1–11.0	38.9	23.0–54.8	25.0	10.9–39.1	19.4	6.5–32.3	19.4	6.5–32.3
**CT60G>A**										
G/G (*n* = 42)	7.3	0.9–13.7	45.2	30.1–60.3	33.3	19.0–47.6	30.8	16.7–44.9	27.4	13.5–41.3
G/A (*n* = 89)	7.2	5.3–9.1	38.2	30.0–46.3	22.0	15.1–28.9	14.4	8.3–20.5	12.6	6.7–18.5
A/A (*n* = 42)	8.3	6.4–10.3	35.7	21.2–50.2	21.4	9.1–33.7	16.7	5.3–28.1	16.7	5.3–28.1

*Median OS and survival rates as estimated from Kaplan–Meier analysis*.

Patients who had received immunotherapy before IPI showed a better survival, although not statistically significant, as compared to patients who did not receive any prior immunotherapy (median OS 10.2 vs 7.3 months). Moreover, the survival pattern according to CTLA-4 genotypes was confirmed also in this subgroup of patients since patients with the G/G genotype had a more favorable survival compared to patients with the G/A genotype for both −1577G>A and CT60G>A SNVs.

## Discussion

The present study assessed the potential role of defined CTLA-4 gene variants in predicting clinical outcome in patients with advanced melanoma treated with IPI. The rationale for this study was based on the assumption that, since CTLA-4 represents a key negative regulator of T cell activation, genetic variants which alter its expression and/or function could affect the interaction of CTLA-4 with IPI and thus its therapeutic efficacy in MM patients. Indeed, we previously provided some evidences of a possible effect of the −1577G>A and CT60G>A SNVs in favoring OS and response to CTLA-4 blockade therapies (20).

In the present study, we further confirmed that the CTLA-4 −1577G>A and CT60G>A SNVs are significantly associated with BOR to IPI treatment.

Multinomial logistic regression analysis of BOR was performed using the following covariates: age and gender, as generic factors commonly used in statistical analyses; presence of visceral metastases as main prognostic factor reflecting the overall clinical status of the patient; LDH levels as well established prognostic factor of survival in melanoma patients; number of therapies given before IPI as factor mostly indicative of the clinical history of the patient. This analysis pointed out a strong decreasing tendency in the relative frequency of responder/progressive patients in comparison to stable patients as a function of the allele A “dose” (0, 1, and 2) in both SNVs. On average, statistically significant reductions of about 70% (G/A vs G/G) and about 95% (A/A vs G/G) were found. In other words, harboring the allele A seems to induce an effect of disease stability.

The homozygous −1577G/G and/or CT60G/G genotypes also appear to favor a long-term survival: patients with these genotypes had more than a twofold higher survival rate at 3 and 4 years compared to patients with the heterozygous G/A genotype.

By contrast, the other −1661A>G, −658C>T, −319C>T, and +49A>G SNVs did not present any relevant role in our analysis.

The precise mechanism by which the G/G genotypes are associated with a favorable BOR and OS in IPI-treated MM patients remains to be determined as no functional data in melanoma patients are available in literature. However, a biological role for the G/G genotype of both −1577 and CT60 SNVs has been reported in healthy individuals being found associated with a significant decrease of CTLA-4 mRNA levels and, consequently, with a reduced CTLA-4 expression at the surface of peripheral blood mononuclear cells (27). Thus, it would be reasonable to hypothesize that a lower CTLA-4 expression level would result in a lower baseline downregulation of Teff cells and reduced interaction with B7 ligands potentially facilitating the blocking capability of IPI. Moreover, the G/G genotype of CT60 SNV might have a prognostic value by reducing the frequency of Treg cells (28) as well as the levels of soluble CTLA-4 isoform (14).

By contrast, the A/A genotype might correlate with higher CTLA-4 expression not only on Teff and Treg cells but also on tumor cells (29) thus facilitating tumor cellular lysis through an IPI-dependent cell-mediated cytotoxicity by Fcγ receptor expressing immune cells, such as monocytes for Treg cells (30) or NK and Tγδ cells for tumor cells (29). A higher cytotoxicity of Treg or tumor cells might balance the higher downregulation of Teff cells harboring the A/A genotype suggesting a possible explanation for the higher proportion of MM patients with irSD among the A/A carriers.

In view of the increasing number of effective drugs available for the treatment of MM, upfront identification of patients who are more likely to fail or to benefit from treatment is a major unmet need. Regarding IPI, only a small subset of patients experience a clinical response to treatment and IPI itself is not devoid of adverse events. This is particularly relevant in the adjuvant setting: IPI 10 mg/kg was recently approved by the FDA as adjuvant treatment for patients with resected stage III melanoma; however, despite a 25% decrease in the risk of relapse compared with placebo in a phase III trial, more than half of the patients treated with IPI experienced a grade 3–4 adverse event and five (1%) patients randomized in the IPI arm died because of drug-related adverse events (31).

Besides IPI, other biological treatments are now available for MM patients. Anti-PD-1 agents have been recently approved by the FDA and patients with tumors harboring the BRAF mutation may be treated with BRAF and MEK inhibitors. Both anti-PD-1 drugs and BRAF and MEK inhibitors have been shown to achieve better response rates and higher median OS than IPI in clinical trials (32–34). Nevertheless, IPI may still have a role as (i) a subsequent therapy in metastatic patients after progressive disease with anti-PD-1 and, if BRAF-mutant, BRAF/MEK inhibitors; (ii) an adjuvant therapy for high-risk stage III melanoma; and (iii) a combination therapy with anti-PD-1 drugs.

In conclusion, our results indicate that the −1577G>A and CT60G>A CTLA-4 variants may have some predictive and/or prognostic role of IPI efficacy in MM patients. However, this concept needs to be validated in further randomized studies comparing patients with similar clinicopathological characteristics treated or not with IPI. In this regard, it is noteworthy that some CTLA-4 SNVs, including CT60G>A, did not show any significant effect in melanoma patients treated with adjuvant interferon alpha therapy (25), thus supporting the hypothesis of some involvement of determined SNVs in CTLA-4-based immunotherapy.

Functional studies are also required to define the mechanisms of action of CTLA-4 gene variants on both Teff cells and tumor cells in IPI-treated melanoma patients.

## Ethics Statement

In accordance with the Declaration of Helsinki, the study protocol was approved by the Ethics Committee of the Liguria Region (CE-IST OMA07.024 emended on 2 January 2011), the Ethics Committee of each participating clinical center and the Scientific Committee of IMI. Written informed consent was obtained from all patients.

## Author Contributions

Study concept and design, study supervision: PQ^1^ and MP. Recruitment and management of metastatic melanoma patients: PQ^1^, CF, CL, PFF, CM, EC, AB, PA, MC, ES, FG, EP, GC, PM, MG^11^, ST, MM, BM, PQ^13^, PF, MG^14^, MR, and FS. DNA extraction, experimental setup, and genotyping: AM, BB, RC, PP, SL, and MR. Acquisition, analysis, or interpretation of data: BD, AM, VF, MR, and MP. Drafting of the manuscript: PQ^1^, BD, FS, and MP. Statistical analyses: BD, VF, and MP. Administrative, technical, or material support: AM, BB, PP, CF, CL, RC, SL, PFF, CM, EC, AB, PA, MC, ES, FG, EP, GC, PM, MG^11^, ST, MM, BM, PQ^13^, PF, MG^14^, MR, and FS. Critical revision of the manuscript for important intellectual content: PQ^1^, BD, BB, PP, MR, FS, and MP. All authors read and approved the final manuscript.

## Conflict of Interest Statement

PQ^1^ is member of the advisory board and consultant of Roche, GSK, Novartis, Bristol, MSD, and Amgen. PFF participated to BMS, Novartis, and Roche advisory boards has served as consultant and received travel support from BMS, Roche, GSK, Novartis, and MSD. PA has/had a consultant/advisory role for BMS, Roche-Genentech, MSD, Novartis, Ventana, Amgen, and Array. He received also research grants from BMS, Roche-Genentech, Ventana, and Array. MM participated to BMS, Novartis, and Roche advisory boards has served as consultant and received travel support from BMS, Roche, GSK, Novartis, and MSD. MG^14^ participated to BMS, Novartis, and Amgen Advisory Boards has served as consultant and received travel support from BMS, Novartis, MSD, and Roche. All remaining authors have declared no conflict of interest.

## Abbreviations

BOR, best overall response; CTLA-4, cytotoxic T lymphocyte antigen-4; EAP, expanded access program; IMI, Italian Melanoma Intergroup; IPI, ipilimumab; MLR, multinomial logistic regression; MM, metastatic melanoma; OS, overall survival; PSQ, pyrosequencing; SNVs, single nucleotide variants.
