# Corrigendum: Association of CTLA-4 Gene Variants with Response to Therapy and Long-term Survival in Metastatic Melanoma Patients Treated with Ipilimumab: An Italian Melanoma Intergroup Study

**DOI:** 10.3389/fimmu.2018.00403

**Published:** 2018-03-02

**Authors:** Paola Queirolo, Beatrice Dozin, Anna Morabito, Barbara Banelli, Patrizia Piccioli, Cristiana Fava, Claudio Leo, Roberta Carosio, Stefania Laurent, Vincenzo Fontana, Pier Francesco Ferrucci, Chiara Martinoli, Emilia Cocorocchio, Angelo Battaglia, Paolo A. Ascierto, Mariaelena Capone, Ester Simeone, Federica De Galitiis, Elena Pagani, Gian Carlo Antonini Cappellini, Paolo Marchetti, Michele Guida, Stefania Tommasi, Mario Mandalà, Barbara Merelli, Pietro Quaglino, Paolo Fava, Massimo Guidoboni, Massimo Romani, Francesco Spagnolo, Maria Pia Pistillo

**Affiliations:** ^1^Department of Medical Oncology, IRCCS AOU San Martino-IST-Istituto Nazionale per la Ricerca sul Cancro, Genova, Italy; ^2^Unit of Clinical Epidemiology, IRCCS AOU San Martino-IST-Istituto Nazionale per la Ricerca sul Cancro, Genova, Italy; ^3^Unit of Tumor Epigenetics, IRCCS AOU San Martino-IST-Istituto Nazionale per la Ricerca sul Cancro, Genova, Italy; ^4^Department of Health Sciences, University of Genova, Genova, Italy; ^5^Unit of Cellular Biology, IRCCS AOU San Martino-IST-Istituto Nazionale per la Ricerca sul Cancro, Genova, Italy; ^6^Intergruppo Melanoma Italiano (IMI) and Department of Internal Medicine, University of Genova, Genova, Italy; ^7^Oncology of Melanoma Unit, European Institute of Oncology, Milan, Italy; ^8^Melanoma, Cancer Immunotherapy and Innovative Therapy Unit, Istituto Nazionale Tumori Fondazione ‘G. Pascale’, Naples, Italy; ^9^Istituto Dermopatico dell’Immacolata IDI-IRCCS, Rome, Italy; ^10^Medical Oncology, Sant’Andrea Hospital, Sapienza University of Rome, Rome, Italy; ^11^Department of Medical Oncology, Molecular Genetics Laboratory, IRCCS Istituto Tumori “Giovanni Paolo II,” Bari, Italy; ^12^Unit of Medical Oncology, Department of Oncology and Hematology, Papa Giovanni XXIII Hospital, Bergamo, Italy; ^13^Dermatologic Clinic, Department of Medical Sciences, University of Turin, Turin, Italy; ^14^Immunotherapy and Cell Therapy, IRCCS-IRST, Meldola, Italy

**Keywords:** CTLA-4 variants, melanoma, ipilimumab, best overall response, overall survival, predictive/prognostic factor

In the original article, affiliation number 9 was wrong. The correct affiliation 9 is “Istituto Dermopatico dell’Immacolata IDI-IRCCS, Rome, Italy”. This error does not change the scientific conclusions of the article in any way.

The original article has been updated.

## Conflict of Interest Statement

The authors declare that the research was conducted in the absence of any commercial or financial relationships that could be construed as a potential conflict of interest.

